# Compositional modelling of immune response and virus transmission dynamics

**DOI:** 10.1098/rsta.2021.0307

**Published:** 2022-10-03

**Authors:** W. Waites, M. Cavaliere, V. Danos, R. Datta, R. M. Eggo, T. B. Hallett, D. Manheim, J. Panovska-Griffiths, T. W. Russell, V. I. Zarnitsyna

**Affiliations:** ^1^ Department of Computer and Information Sciences, University of Strathclyde, Glasgow, UK; ^2^ Centre for Mathematical Modelling of Infectious Disease, London School of Hygiene and Tropical Medicine, London, UK; ^3^ Department of Computing and Mathematics, Manchester Metropolitan University, Manchester, UK; ^4^ Département d’Informatique, École Normale Supérieure, Paris, France; ^5^ Datta Enterprises LLC, San Francisco, CA, USA; ^6^ MRC Centre for Global Infectious Disease Analysis, Imperial College London, London, UK; ^7^ Technion, Israel Institute of Technology, Haifa, Israel; ^8^ The Big Data Institute and the Pandemic Sciences Institute, Nuffield Department of Medicine, University of Oxford, Oxford, UK; ^9^ The Queen’s College, University of Oxford, Oxford, UK; ^10^ Department of Microbiology and Immunology, Emory University School of Medicine, Atlanta, GA, USA

**Keywords:** epidemics, immune response, process calculi, multi-scale modelling, COVID-19

## Abstract

Transmission models for infectious diseases are typically formulated in terms of dynamics between individuals or groups with processes such as disease progression or recovery for each individual captured phenomenologically, without reference to underlying biological processes. Furthermore, the construction of these models is often monolithic: they do not allow one to readily modify the processes involved or include the new ones, or to combine models at different scales. We show how to construct a simple model of immune response to a respiratory virus and a model of transmission using an easily modifiable set of rules allowing further refining and merging the two models together. The immune response model reproduces the expected response curve of PCR testing for COVID-19 and implies a long-tailed distribution of infectiousness reflective of individual heterogeneity. This immune response model, when combined with a transmission model, reproduces the previously reported shift in the population distribution of viral loads along an epidemic trajectory.

This article is part of the theme issue ‘Technical challenges of modelling real-life epidemics and examples of overcoming these’.

## 1. Introduction

Our starting point in this paper is work by Hellewell *et al*. [1] where they fit a descriptive statistical model to empirical data [2] describing cycle threshold (Ct) values for PCR tests of healthcare workers in England. Rather than describing the data, we aim to get at the mechanism behind it. We begin by asking: what is the simplest, biologically reasonable, process that is sufficient to produce such data? Our answer to this question also produces insights into a possible biological basis for over-dispersion of infections [3] and, when an infection transmission process is added, the observed shifts in distribution of Ct values during the rising and falling of an epidemic [4].

The method that we use builds upon previous theoretical work in applying techniques first developed for molecular biology [5,6] to epidemics [7]. These techniques facilitate two important features: *modularity* and *compositionality*. Modularity means that it is possible to create self-contained models, in this case of immune response and transmission that can be individually calibrated and studied. Compositionality, in our context, means that one can create larger and more complex models by combining, in a straightforward and flexible way, the sets of rules associated with different and simpler models [7,8]. Complex and non-trivial behaviour of large models can be obtained by combining the instructions corresponding to simpler models with simple behaviours [7,8]. This is important for several reasons. Some operations, particularly calibration or fitting to data, are both computationally expensive and critical for real-world applications. We would like, so far as possible, to do these operations once and reuse them. Moreover, as discussed in [7], the compositionality offered by the rule-based approach has advantages in terms of syntactic complexity and scalability of the model description compared to alternative methodologies (e.g. ODEs).

Beyond the immediate subject-matter of this paper, epidemic trajectories strongly influence and are influenced by processes at several spatial and temporal scales: biology and physiology of the hosts, behaviour and individual choices, policy choices and decision-making, and economic environments to name a few. All of these topics require sophisticated modelling efforts in their own right and a substantial amount of domain expertise. Any strategy for understanding the interplay of these processes that relies on a monolithic approach for modelling is unlikely to be feasible. A modular approach that breaks the large system down into components individually modelled at appropriate scale [9] and then composes them into a model of the whole is much more likely to be effective because the individual components can be small enough to be tractable and enable domain experts to focus their expertise on them. It is equally important that the composition of such models are well defined if we are to understand the properties of the combined model. We use a method of doing this that builds on a solid foundation of abstract mathematics and category theory [5,10,11] to ensure that this is the case, as well as properly representing the biological and epidemiological processes.

Multi-scale models of within- and between-host transmission exist and are recognized as an important line of research [12,13], and attention has been given to the possibility of composite models for this purpose [14]. Nevertheless, the bulk of infectious disease models [15–17] are not formulated in ways amenable to composition, perhaps because methods for defining interfaces between models and the interaction of timescales and concepts of discrete and continuous time remain poorly understood. This remains an important challenge, however progress can be made. There is a long-standing recognition of the need for modular composable models and there are well-developed efforts in this direction in synthetic and systems biology [18–25].

The remainder of this paper is structured as follows. We first briefly introduce rule-based models in general. We then give our model of adaptive immune response and show the implications for the expected distribution of viral load in terms of time since infection that follows from it. Next, we give the model of transmission and how it is coupled to the immune model and show how the distribution of viral load can be expected to vary according to an epidemic trajectory. Finally, we discuss some limitations and future research directions.

## 2. Compositional modelling with rules

The model that we present here, and its constituent sub-models, are formulated as rules. Sets of rules can be composed together; rule-based modelling is a particular style of compositional modelling. Informally, a rule is the way states of the model change. A rule is a structure with a left-hand side L, a right-hand side R and a rate, k. L is interpreted as a pattern that matches some part of the system, for example, people with a given characteristic or state or even relationships. R is an instruction for how the configuration should be changed when that pattern matches; the action of a rule is to rewrite the configuration of the system so that it evolves over time. In a single simulation step, a single match, or embedding, is chosen from all possible embeddings E(L) in the graph representing the state of the system, and this embedding is replaced according to the instruction. Thus, a rule encodes many possible transitions (one for each possible embedding) in a continuous-time Markov chain (CTMC) where the state-space is all possible graphs. This Markov chain can be simulated with the usual method where the propensity of a rule to operate is given by the number of embeddings, and the rate, p=k|E(L)|. It is possible to sample trajectories from this CTMC exactly without materializing the entire state-space or employing moment-closure techniques [6]. It is also possible to systematically produce ordinary differential equations for the moments of the CTMC, but they are rarely feasible to use in practice for any but the simplest^1^ models because of the high dimensionality involved.

The algebra of rules that governs how rules compose is an advanced topic [10], but it is easy to obtain an intuition about how it works with a version of Gillespie’s algorithm [26]. Suppose that we have two rules, $r_{1}$ and $r_{2}$. To perform one simulation step for a model with only one rule, we choose one match uniformly at random of the left-hand side of the rule in the system state, replace it with the right-hand side and advance time by an exponentially distributed amount with rate given by the rule’s propensity. Composing the two rules, we perform a step by first choosing which rule to use with probability proportional to their propensities. Having chosen a rule, we then choose a match uniformly at random, do the replacement, and advance time as above with a rate of the total propensity of all rules. This is exactly analogous to simulating a single chemical reaction, except that the ‘match’ and ‘replace’ operations are more complex because they involve searching in and manipulating a graph as opposed to counts of chemical species. Fortunately, we have software that can do this efficiently [6].

**Figure 1.**
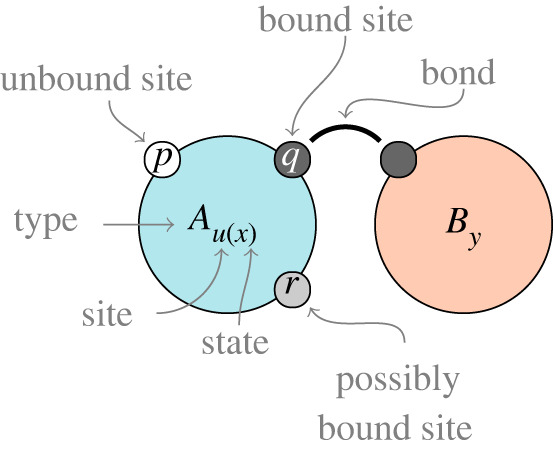
Illustration of an agent pattern with features identified. The pattern consists of two agents, A and B. Agent A has four sites: p is not bound, q is bound to a site on agent B, the binding state of r is unspecified (and normally would not be shown) and u has an internal state x and does not participate in binding. Agent B has two sites with names elided, which can often be done to avoid visual clutter where there is no risk of ambiguity. (Online version in colour.)

In the κ language [5,6], the configuration of the system is described as a site-graph where vertices are called *agents* (by analogy with the reagents in chemistry, not to be confused with the agents of agent-based models, though some parallels exist), that have *sites* which can have internal *state* and edges are *bonds* between sites of different agents. The biochemical heritage of the formalism is evident from this nomenclature. Figure 1 shows an example agent pattern (e.g. the left-hand side of a rule) pictorially. It consists of two agents, A and B. Both have several binding sites which can be in an unbound (p) or bound (q) state. For patterns, we may not care whether a particular site is bound or not, normally such sites would not be mentioned in a pattern, but when we wish to explicitly depict it, we do this with a half-shaded site (r). A has a site u with internal state x. Where there is no risk of confusion, we may drop the name of the site, as with B which has a nameless binding site and a site with state y. For a thorough explanation of this arrangement in practice in its original setting, the reader may wish to consult Boutillier *et al*.’s original article [6], as well as our tutorial with examples on the application to infectious diseases [7].

We will use several agent types in the models given below. Agents of type P represent people—chosen rather than I for individual to avoid confusion with individuals in an infectious state as appears in many susceptible, infectious, removed (SIR) style models. Elements of immune response are represented by agents bound to a person, B for B-cells, A for antibody populations. The viral population within a host is represented as an agent of type V.

Throughout this paper, in the pictorial representation of rules, we use colour suggestively. All the necessary information to understand the rules is contained in the agent and site labels, however we will typically use colour in addition. For example, a susceptible individual is shown in blue, 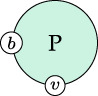, an infectious one in red, 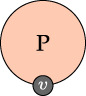 and a removed individual in yellow, 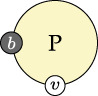. Note that in all of these cases, the site patterns are sufficient to distinguish the disease progression state of the individual and the colours simply reinforce this visually.

## 3. Adaptive immune response

While we use a simplified model here, the general approach can be extended to more complex models of immune response with relatively little effort. We give the model in mathematical form, with explanatory narrative. A methodological observation is that, though we use shape and colour for visual interest, there is an exact correspondence between what is depicted and the machine-readable computer code used for simulation reproduced in appendix A along with some discussion of implementation details in appendix B.

### (a) Viral load

The entire within-host response is driven by replication of the virus. We do not represent individual virions in this model and simply track the size of the virus population. We track this with a counter that represents the logarithm of the population size. Replication is captured simply by incrementing this counter,
3.1
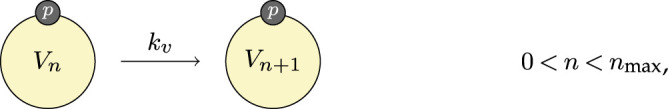

up to some maximum. In principle, uncontrolled replication should proceed up to the carrying capacity of the host. For simplicity, we represent this process as simple exponential growth with a maximum limit rather than logistic growth.

### (b) Initial activation

The peak of viral replication is usually controlled by the innate response together with target cell limitation and further clearance of the virus is controlled by CD8 T-cells and antibodies [27–29]. In this simplified model, we do not represent the innate response, and we consider notional antibodies as generalized effectors providing the only mechanism for clearance of viral particles. We begin with the activation of B-cells in an infected individual bound to a virus population,
3.2
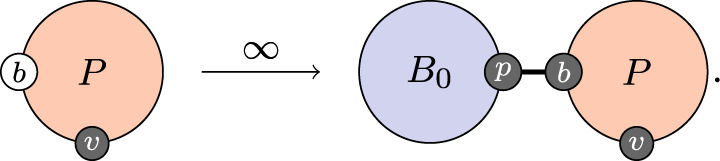

On the left-hand side, the individual has no activated B-cells, and on the right-hand side, they acquire a population of activated B-cells. The subscript on B indicates that all B-cells in this population are naive: they have very low affinity for viral proteins. This process is interpreted as *allocation of a population* of B-cells and not production of the cells themselves. This is somewhat of a computational fiction and is an artefact of the simplifying choice as with the virus population to not track individual cells, but only their count (or in this case, their affinity). The process therefore proceeds immediately, at infinite rate.

The function of B-cells is to produce antibodies. Again, as with the virus population, we do not track individual antibody proteins, we simply track their number, again logarithmically. The second process in the activation of immune response is,
3.3
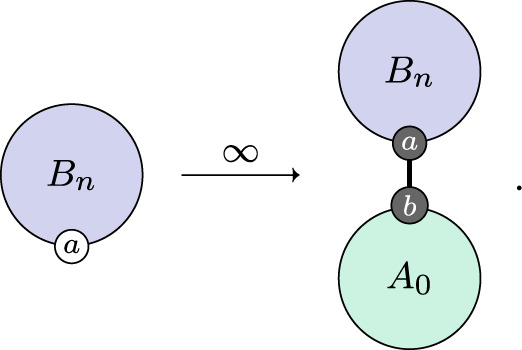

As with the activation of B-cells, this process is interpreted as allocation of a population of antibodies, not the production of antibodies themselves. Antibody production is a separate rule (equation (3.5)). As with the allocation of a B-cell population, the allocation of an antibody population proceeds immediately.

**Figure 2.**
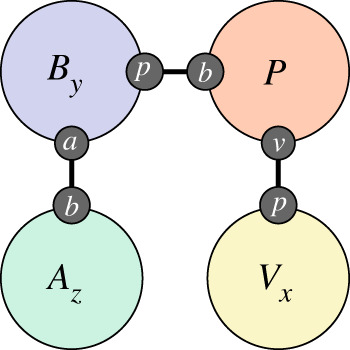
Fully active immune response with viral load x, B-cell affinity y and antibody count z.

Once the immune response has been activated, the configuration of the within-host state for an individual is as depicted in figure 2. The individual is bound to a virus population of a certain size, x (the process by which this happens is exogenous to the immune model), the individual is also bound to a B-cell population with average affinity y and these B-cells are bound to an associated antibody population with count z.

### (c) Immune dynamics

Now we can describe the dynamic processes of the adaptive immune response. They begin with affinity maturation. The process of affinity maturation is only partly understood. Virus-specific B-cells, initially with low affinity, are recruited into special structures called germinal centres (GCs), where their affinity maturation occurs. In GCs, B-cells get stimulated through their B-cell receptors by the antigen and undergo the rounds of proliferation with somatic hypermutation (SHM) of their immunoglobulin genes. The cycles of SHM coupled to selection for antigen binding lead to the generation of B-cells of higher affinity. During the affinity maturation process, a fraction of B-cells further differentiate into plasma cells that begin to secrete virus-specific antibodies [30]. SHM continues, and the antibodies produced by the B-cells are iteratively refined and better tuned for binding to the virus. We elide the detail of this fascinating process and treat it phenomenologically at a higher level of abstraction. We reason that in a well-mixed environment, the chance of a B-cell encountering a compatible virion is proportional to some function of the virus population and write,
3.4
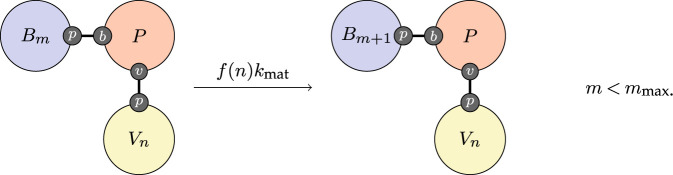

For simplicity, we take f(n)=n and obtain $k_{\text{mat}}$ by calibration.

By similar reasoning, antibodies are produced at a rate that we take to be proportional to the affinity,
3.5
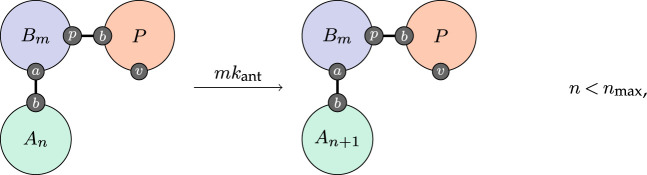

and likewise obtain $k_{\text{ant}}$ by calibration. Note that the P agent appears in this rule with an explicitly bound virus population. This is because virus-specific B-cells populations arise and are activated only in the presence of viral proteins; if there are no such proteins, negligible amounts of antibodies (none in our model) are produced.

The action of antibodies is to disable or neutralize virions. We follow a similar pattern and suppose that this happens at a rate proportional to the antibody population,
3.6
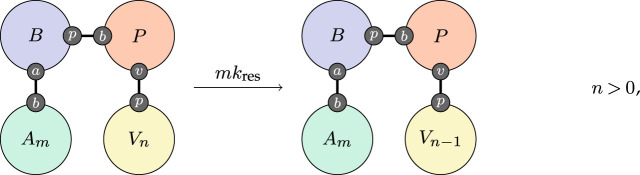

with the constant of proportionality $k_{\text{res}}$ again obtained by calibration.

Finally, recovery is said to happen when the virus population drops sufficiently low,
3.7
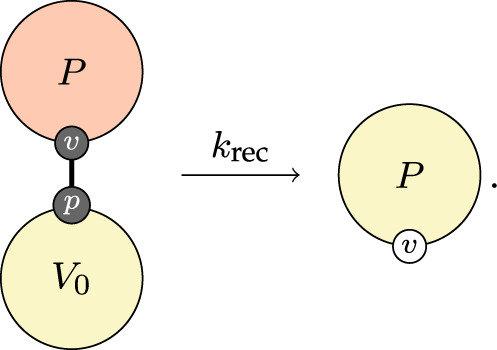

Though we do not need to specify it in this rule, the recovering individual will still have a population of B-cells with some affinity and its associated antibody population. We do not have a rule that severs those edges, nor do we have a rule that can decrease affinity. This provides a mechanism for immune memory. If a previously infected individual is exposed to the virus again, they may have a population of antibodies that can immediately dispatch it and if they do not, their B-cells can produce them.

Finally, we include a simple rule very similar to the above for waning, where the antibody population decreases slowly when the individual does not have a virus population,
3.8
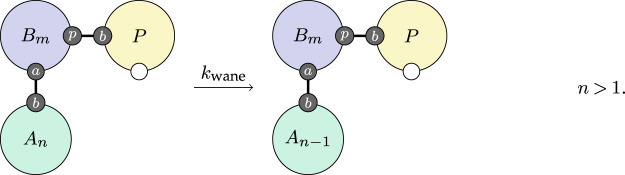


### (d) Connection to compartments

In epidemiological models, it is traditional to subdivide the population into susceptible individuals, removed individuals, and one or more compartments of individuals in different states of incubation or infectiousness. Even with individual- or agent-based models, this classification of individuals is commonly used. There is a natural way to make the connection to these concepts simply by counting configurations of individuals and their immune response. This is done by defining *observables* that count the number of embeddings of a pattern similar to the operation of the left-hand side of a rule,
3.9
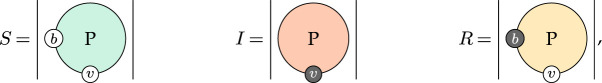

where the vertical bars are used in the sense of cardinality of a set—the set of embeddings induced by the patterns. A susceptible individual is one who has no virus population and no established immune memory. An infected or infectious individual has a virus population regardless of the state of their immune response and a recovered or removed individual has no virus population but does have established immune memory. These correspondences of course only give a coarse picture of the expected dynamics: they do not consider the robustness of the immune memory or the degree of infectiousness. This coarseness is the reason that, if we were to work at the level of compartments (i.e. high-level disease progression states whether in a compartmental model or otherwise), we would be required to formulate processes in terms of complicated distributions chosen either by hypothesis or empirically. Here, we do not need to do that because we have access to a fine-grained account of adaptive immune response. It is nevertheless useful to have a view of the dynamics of this model that is comparable to the more common representation.

### (e) Empirical validation

It is easy to see that viral load—the quantity of virus carried by a host—must vary over the course of the disease. At the time immediately prior to infection, there is no virus present. At some future time after the infection has been cleared, there is likewise no virus present. At some time in between there must be some non-zero amount of virus present because that is the meaning of infection. Therefore, viral load must have been increasing for some of that time, and decreasing for some of that time and it must have had a maximum for some time between these phases. While there is no *a priori* guarantee that there must be only one occurrence of each of these phases, the simplest behaviour would be a single increasing phase, a peak and a decreasing phase. This is precisely what we observe empirically through assays that are sensitive to the presence of virus itself (e.g. PCR) or viral proteins (e.g. antigen) [1,31–33]. Furthermore, because the various immune processes that eventually suppress the population of virions take time to develop, any increase in antibody products must lag behind an initial increase in viral load, again, precisely what is observed with antibody assays [31–33].

**Figure 3.**
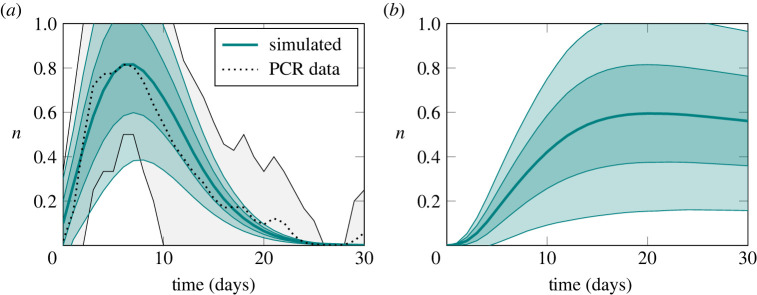
Within-host immune dynamics sampled from a single simulation of a population of 10 000 individuals. The system is fit to PCR response data response as reported by Hellewell *et al*. [1]. Underlying processes of virus replication, affinity maturation, antibody production and virus neutralization reproduce the characteristically asymmetric viral load curve (*a*) and an antibody response curve (*b*) that lags viral load. The vertical axis in both figures is measured in arbitrary logarithmic units. The uncertainty envelopes correspond to 1 and 2 s.d. from the mean. (Online version in colour.)

The adaptive immune response model reproduces the above observations from the underlying processes. We calibrated the model with the approximate Bayesian computation (ABC) method using a root mean square distance relative to the mean PCR test response for SARS-CoV-2 infection as reported by Hellewell *et al.* [1] based on data from a study of health workers in England [2]. The fitting procedure is computationally expensive, however, it need only be performed once: the calibrated immune model stands on its own and for many uses, it can be combined with other models without the necessity to repeat this expensive procedure. The resulting viral load and antibody response curves for a single simulation of a population of 10 000 individuals are shown in figure 3 with uncertainty envelopes corresponding to 1 and 2 s.d. from the mean, and the reference data together with its 95% credible interval is also shown in figure 3*a*. Because individuals are non-interacting, there is no salient distinction between a single simulation of 10 000 individuals and 10 000 simulations of a single individual.

The measurement scale used in figure 3 is logarithmic and truncated. In the adaptive immune response model, the populations of virions and antibodies are both represented as an integer, n. This integer n is interpreted as the logarithm of the size of the population. The justification for this interpretation is the nature of testing with PCR and laboratory assays. Quantitative results are obtained by either successive dilutions (titrations) or by successively culturing under conditions that permit exponential growth. The cycle threshold value reported for PCR tests, for example, is the number of growth cycles required to detect RNA from the virus, and can be interpreted (with a change of sign, up to a constant factor) as the logarithm of the initial amount of RNA. Similar reasoning applies to the titres reported from laboratory assays for antibodies or antigens. In both cases, a positive or negative result is simply a statement that a threshold value has been passed. The measurement scale n that we use is truncated for practical reasons at a maximum value $n_{\text{max}}$. The rates at which various processes in the model occur are expressed in terms proportional to n for the various entities involved. This interpretation, together with the fitting procedure as described, rests on the following assumption connecting the mechanistic model to the empirical test data: *the rate of positive test results is proportional to the logarithm of the viral population.*

### (f) Viral load distribution

**Figure 4.**
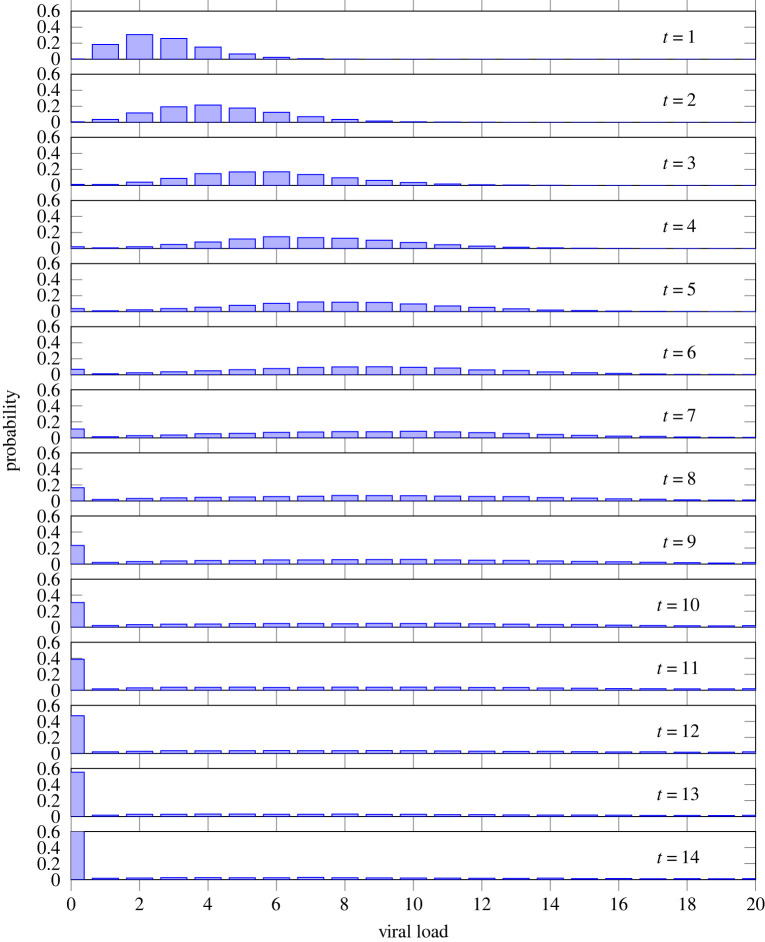
Timeseries of probability distributions of having a viral load on a scale of 0 to $n_{\text{max}}=20$, from the same simulation of 10 000 individuals as figure 3. By day 14, 80% of viral load is concentrated in approximately 20% of individuals. (Online version in colour.)

Figure 4 shows data from the same simulation of 10 000 individuals. All individuals are infected at the beginning of the simulation. It depicts the probability distributions of an individual to have a particular viral load as measured on the scale that we have defined, from 0 to $n_{\text{max}}=20$. We observe a wave of probability starting with a certainty that all individuals have a viral load n=1, with viral load increasing as time progresses. After about 10 days, a significant result appears: a long-tailed distribution where an ever-smaller proportion of the population has a large viral load.^2^ This is significant because it suggests a biological basis for the phenomenon of over-dispersion in infectiousness, typically captured in infectious disease models by asserting an appropriately parametrized Gamma distribution of infectiousness [3]. By the end of this 14-day sequence, 80% of viral load is concentrated in approximately 20% of the population and by day 21 this has shrunk to 3%. On average, over the entire 30-day period of the simulation, 80% of the viral load is concentrated in 21% of the population. Teasing out the implications of these results can shed some light on the phenomenon of superspreading individuals. On the one hand, increasing viral load may increase the chance of an individual transmitting virus in any given encounter. On the other hand, increasing viral load may also increase the chance of developing symptoms, to the extent that the individual would be more likely to self-isolate. Thus the chance of encounters occurring would diminish. The product of these two factors gives the overall chance of transmission. The interplay between these two factors: transmissibility upon a given encounter (increasing with viral load) and chance of encounter (decreasing with viral load) may result in the overall chance of transmission being a concave function of viral load, with a peak at an intermediate viral load. Factors that increase the chance of encounter at any given viral load (such as lack of paid sick leave from a public-facing job) would shift the peak of the function right (to a higher viral load). However, if an individual with a large viral load is present in a situation where transmission is likely, then it is reasonable to expect that a superspreading event may occur [34].

In passing, we observe that the processes in the adaptive immune response model are more than strictly required to reproduce this kind of long-tailed distribution of viral load. It is possible to show that a simpler model where load simply increases incrementally and then decreases, with the turning point chosen stochastically will produce a similar result. Such a simple model, however, lacks biological realism and fails to provide a causal or mechanistic account of the phenomenon.

## 4. Transmission dynamics

Unlike immune response which occurs within individuals, transmission is a population-level process that occurs between individuals. With the above formulation in hand, a simple model of transmission is very natural,
4.1
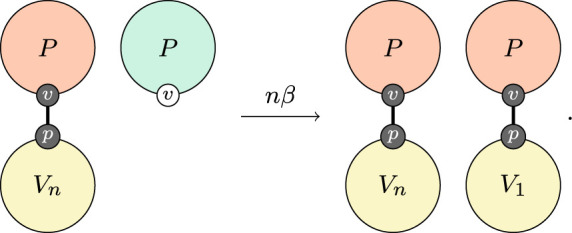

This says that an individual with some virus encounters an individual without the virus (this implies well-mixed, mass action kinetics) and as a result of that interaction, the second individual gets a small virus population.

Note in particular that it is not necessary to speak of any previous infections experienced by the second individual. Nothing corresponding to immunity is required here because *if* an individual with established immune memory is exposed to the virus, *then* their immune response will simply be very rapid and they will clear the virus quickly. There is no need to impose an *a priori* restriction on individuals being exposed more than once or to assert a sharp distinction between individuals that are ‘immune’ and those that are not.

**Figure 5.**
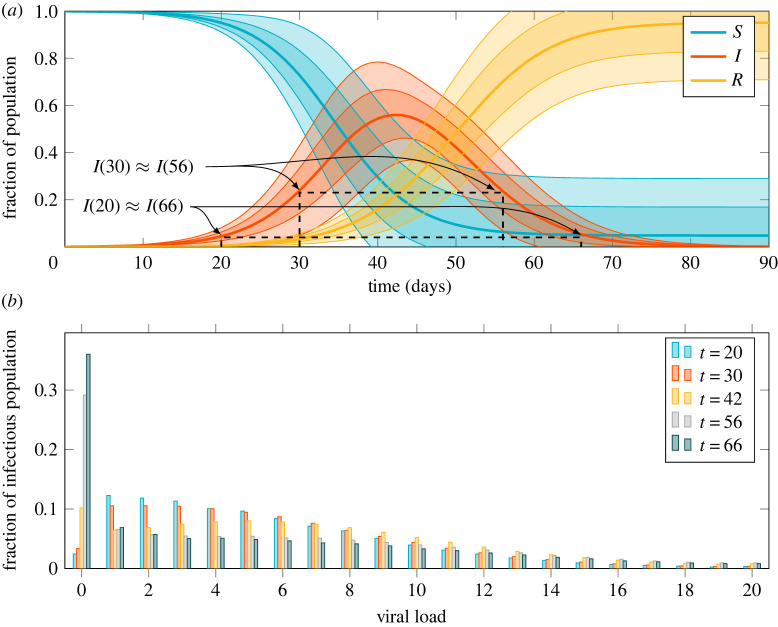
Epidemic curve and viral load distributions for a rising, stationary and falling epidemic. (*a*) Epidemic curve showing the susceptible (S), infectious (I) and removed (R) observables for a population of 10 000 individuals calibrated for a reproduction number of 3. Envelopes show 1 and 2 s.d. over 128 simulations. Marked on the graph are two pairs of time points where the mean number of infectious individuals are equal as the epidemic rises and falls. (*b*) Viral load distribution at different points of the epidemic trajectory showing a rising t∈{20,30}, stationary t=42 and falling t∈{56,66} epidemic. Viral load in arbitrary logarithmic units. The probability masses of distributions are shifted to the left (lower viral loads) for a rising epidemic and the distribution for a falling epidemic is in fact bimodal with most infected individuals on the point of recovery but a significant number with slowly decaying high viral loads. (Online version in colour.)

Figure 5*a* shows the epidemic curve produced by the combined transmission and immune model according to the observables (cf. §3d) corresponding to the standard epidemic model compartments. The rate parameter β in equation (4.1) is fit so as to produce an epidemic with a basic reproduction number $R_{0}=3$, typical of the ancestral strain of SARS-CoV-2 in a Western European setting. Determining a precise value for $R_{0}$ and hence β is setting-specific, strongly affected by behaviour, and of only peripheral interest here. Two pairs of time points are marked on the figure, corresponding to the times at which, on average, the same number of individuals are infected as the epidemic rises and falls. The first pair, t∈{20,66}, is when a small number of individuals are infected and the second, t∈{30,56} when a much larger number are. Finally, we note that the epidemic is stationary (i.e. peaks, is neither rising nor falling) around t=42.

Let us look at the viral load distributions at these chosen time points in more detail, figure 5*b*. We see that the distributions differ substantially. For a rising epidemic (t∈{20,30}), the bulk of the probability mass is shifted towards the left, indicating a lower viral load. As individuals become infected faster and faster, there are more individuals who are at an early stage in the course of their infection. Recall from figure 3*a* that we expect a peak viral load perhaps a week after initial infection and then a slow decay over the subsequent several weeks. On the other hand, for a falling epidemic (t∈{56,66}), the probability distribution is bimodal. Much of the probability mass is concentrated in the lowest viral load as many individuals are on the point of recovery. However, a significant amount of probability mass is present in high viral loads reflecting individuals with developed infections and slowly decaying viral loads. A similar effect was noted by Hay *et al*. [4] who showed that rising and falling epidemics can be distinguished by looking at the distribution of cycle threshold values in PCR tests and that this can, in fact, be used to estimate the time-varying reproduction number R(t) in a population. We recover this result as a consequence of the immune dynamics in the model.

## 5. Discussion

Previous work on models, both across domains and for infectious disease modelling specifically [35], have suggested that there are various features which make models more useful for different tasks. For infectious disease modelling, useful features include predictive accuracy, ability to effectively model historic behaviour and predict the outcomes of future interventions in a way that reveals causality, flexibility to update both the model structure and calibration of parameters as new information becomes available, computational tractability, and ease of explanation to policy-makers and the public. The approach presented here has significant advantages across many of these desiderata though there is also still work to be done. Our emphasis on *modularity* and *compositionality* facilitates the flexibility to update model structure: individual rules or entire sub-models can be changed or substituted locally, without requiring global changes. Indeed, it is possible to analyse the dependencies between rules to determine precisely the extent to which modifications to one part of the model may require modification to other parts. The modular structure means that it is often possible to calibrate sub-models individually, saving the great computational expense of performing this operation for all parameters, which is often necessary for monolithic models.

Additionally, this modular and compositional approach facilitates multidisciplinary collaboration. Infectious disease modelling, immunological modelling, modelling of behaviour and economies and supply chains all require a substantial amount of domain expertise and each has a substantial literature of sophisticated models particular to that field. By encapsulating the models and paying attention to the (often unclear or fuzzy) boundaries between them, there is hope for constructing larger, richer models without the tight coupling required by monolithic approaches. Not only that, but we can mix and match. Perhaps a particular immune model is computationally intensive and the cost outweighs the benefit for the question at hand; it can simply be replaced with a simpler one with a compatible interface. Quantitative models of behaviour are difficult to calibrate because data to relate them to ground truth are scarce. This becomes a surmountable obstacle because one can simply use several; such models are simply assumptions of dynamics about which our knowledge is limited so we can evaluate the entire system under different sets of assumptions ranging from simple to very sophisticated. The ability to slot these different models into the same context can allow us to take advantage of decision-making frameworks that incorporate multiple predictors, each of which individually may be ‘weak’ (i.e. low-confidence). *Boosting* is an example of such an ensemble method [36,37]. The structure described is that of *Open Systems*, a concept that dates at least to von Bertalanffy’s work in 1950 [38]. Recent advances in mathematics for interacting systems [39] stochastic rewriting systems [11] (of which the models presented in this paper are examples) and specific kinds of open systems such as Petri nets [40,41] and economic games [42,43] are placing these ideas on solid theoretical ground for computation. By leveraging these theoretical advances, we stand to make great practical benefit in multi-scale and multi-disciplinary modelling. Even more ambitiously, such approaches allow representations used in sets of models to scale from physics to chemistry to biology to epidemiology, pharmacology, and more. This is consistent with similar approaches being taken in systems biology [44], pharmacology [45] and (bio)chemical engineering [46]. Of course, any one model or set of models will occupy only a small part of this spectrum; however, through composition and selection of models, many types of interdisciplinary and trans-disciplinary modelling approaches become possible, enabling us to address an immense variety of questions.

There are numerous challenges to fully realizing this dream. A major obstacle is that there is, at present, no developed theory of how the behaviour of one model influences the parameters of others—though some work in other fields is beginning to address this [47]. This challenge is reflected in our discourse above where we explicitly choose how rates (of transmission, say) depend on values (e.g. viral load) that are produced as a result of a different model. This pattern, where the state of the system as described by one process influences the parameters of a different process, is very common and, at present, must simply be handled manually. A promising approach is that of Open Games [42,43,48] where the composition of games is defined for *all possible* utility functions—essentially parametrization of the games. We could imagine Petri nets with rates or more general kinds of stochastic rewriting systems taking a similar approach where their composition defined for all possible rate functions with specialization to particular choices of rate function done *post hoc* once the complete model is assembled. The combination of Open Games and Open Stochastic Rewriting Systems is potentially very powerful. Game theory is a unifying framework that can be used to underpin statistics [49], machine learning [50,51], microeconomic theory [52] and the behavioural sciences [53], and is already used in a more specialized way in epidemiological modelling [54]. The Open Games formalism for compositional modelling supports combining sub-models that may include games at different levels; for instance, within-host models where the agents are immune cells and virions within an organism, with between-host models where the agents are various organisms within a social milieu. This speculation suggests a path for incorporating epidemic models (which may themselves include game theory) into more overarching game-theoretic accounts in a principled way.

Another challenge is that the formulation of these models in the standard κ calculus permits only well-mixed interactions and site-graphs. In other work [55], we have extended the language to support more general graphs of the kind that are needed for epidemic models on networks, but a further extension to capture explicit hierarchical notions of space as suggested in work on stochastic bigraphs [56] would be beneficial.

The model of adaptive immune response given here is greatly simplified and we have provided no more than a vestigial model of the innate response. For COVID-19, it appears that the innate response plays an important role in whether an individual will recover after the initial acute phase of the disease or go on to develop severe disease [57]. The precise reason for this is not known. Equally, it is an open question why some individuals appear to recover completely and others develop persistent and varied symptoms known as ‘long COVID’ [58]. These persistent symptoms may be rooted in immune response or could have a physiological explanation. In both cases, there is hope that more sophisticated models of immune response and physiology could provide the necessary insight. Beyond these immediate questions, multi-scale, multi-system models are relevant not only for understanding population-level dynamics but for drug discovery and precision treatment of individuals. Modular and compositional modelling techniques such as we demonstrate here provide a method for taming the substantial complexity involved.

## Data Availability

Source code is available at: https://git.sr.ht/~wwaites/immune-transmission and some implementation details are included as appendix B. Calibration data and sampled trajectories are available at: https://datashare.ed.ac.uk/handle/10283/4056.

## Footnotes

1 A typical stratified infectious disease model might have, when rendered as a system of ODEs, at most a hundred dimensions and perhaps a thousand terms. The simple rule-based model that we present here, though small, corresponds to a system of ODEs with nearly ten thousand dimensions and nearly a hundred million terms.

2 While this manuscript was undergoing revisions, we happened to observe this heterogeneity in the wild. The corresponding author of this paper contracted SARS-CoV-2 and unfortunately infected another member of his household. Both indivudals conducted daily lateral flow tests which are known to respond with increasing intensity to viral load. While this does not go beyond the level of anecdote, the test series is nevertheless interesting and shows the first individual with sufficiently high viral load to produce a strong positive test result for more than 10 days and the second cleared the virus rapidly. A photograph of the test series is reproduced in appendix C for interest.

## Appendix A. The combined model

This model contains all of the rules that are described above, in machine-readable form.

## Appendix B. NetABC and the stratified Kappa

**(a) The NetABC software package**

The KaSim simulator has a single, well-defined purpose: to simulate models written in the Kappa language. Though the most interesting part of modelling in the style that we present in this paper is formulating a model such that it corresponds closely to a narrative description of the biological processes, there are various other tasks required for a complete work, namely fitting the model to data, sampling many trajectories and producing numerical summaries to produce figures. These necessary extra tasks are the purpose of the NetABC software.

NetABC^3^ is written in Python and is designed to be used as a command line tool. It has various sub-commands that work together to set up the simulation environment with initial conditions, fit data and produce output. NetABC supports several other model types in addition to models written for the KaSim simulator such as network processes with the Epidemics on Networks package, an experimental dialect of Kappa that runs on networks, Covasim models, etc. Those other model types (and the extra software that they depend upon) are not needed to run a plan Kappa model.

Below is an example command line for fitting data, lightly modified for brevity from the Makefile in the data/ subdirectory of the software distribution underpinning this paper. It consists of (i) fixing constant parameters, (ii) specifying a model, (iii) specifying storage for the fitting process and (iv) specifying a measure and prior distributions for fitting.

The input for this command is the model (contained in the file immune-transmission.ska), reference data to fit against (contained in the file empirical_samples_pcr_detection.csv), fixed parameters and prior distributions for parameters. The output of this command is the file immune.db which contains the posterior distributions of the parameters. This file serves as input to the next step which is to conduct a simulation with parameters drawn from the posterior,

Recall that this particular simulation is of 1000 non-interacting individuals: conducting a single such simulation is equivalent to conducting 1000 simulations of a single individual.

The output of this simulation is stored in the immune.h5 file which is in the standard HDF5 format, commonly used for numerical data in high-performance computing settings. NetABC also has commands for generating TSV files from HDF5 storage to facilitate plotting.

**(b) The stratified Kappa**

NetABC also contains a pre-processor that is run when reading in a Kappa model for the KaSim simulator. This provides extra flexibility for features that are not currently available in KaSim. There are two directives that are placed in blocks. The first allows inclusion of Python language code,

The code in this block is executed once. The second allows iteration of the code contained in the block, with substitution of variables,  iterate.

The original purpose of these extensions was to allow expression of stratified epidemic models where rules of identical form must be repeated with different rates for interactions of different strata in the population. For example, suppose there are two groups with a contact matrix. This might be expressed as,

This model fragment is equivalent to,

For the present work, we make use of these facilities to work around a missing feature in KaSim. We make heavy use of counters, however while it is possible to write rules of the form,

which says ‘decrement the count value at rate k if it is greater than 1’, it is not possible to write,

to increment the counter if it is less than or equal to 10. This feature is likely to be added to the KaSim dialect of Kappa in future, though for now, we work around it by using the preprocessor to generate extra rules,

resulting in,

This approach is somewhat inefficient—in general, it is recommended to have as few rules as possible for a model—but it is not otherwise a serious problem since the semantics are clear.

We similarly generate separate observables for each counter value by iteration,

For completeness, the plain Kappa version of the model is included in the kappa/ subdirectory of the software distribution accompanying this paper alongside the more succinct stratified version. The plan version can be produced with the netabc stratify command.

## Appendix C. Daily lateral flow test results of two individuals

Two series of daily lateral flow tests. The series on the right begins with a strong positive test result on 22nd of February 2022. Positive results continue until the first negative result on 3rd of March. This individual was likely infectious before the series began. The series on the left is initially negative and registers a weak positive on 26th of February followed by two strong positive tests and a weak positive before testing negative on 1st of March.
